# Age- and Sex-Dependence of Dopamine Release and Capacity for Recovery Identified in the Dorsal Striatum of C57/Bl6J Mice

**DOI:** 10.1371/journal.pone.0099592

**Published:** 2014-06-12

**Authors:** Emma Arvidsson, Thomas Viereckel, Sanja Mikulovic, Åsa Wallén-Mackenzie

**Affiliations:** 1 Department of Neuroscience, Unit of Functional Neurobiology, Uppsala University, Uppsala, Sweden; 2 Department of Neuroscience, Unit of Developmental Genetics, Uppsala University, Uppsala, Sweden; University of Chicago, United States of America

## Abstract

The dorsal striatum is the main input structure of the basal ganglia and the major target area of dopaminergic projections originating in the substantia nigra *pars compacta*. Heavily involved in the regulation of voluntary movement and habit formation, this structure is of strong importance in Parkinson's disease, obsessive-compulsive disorder, Tourette's syndrome and addiction. The C57/Bl6J mouse strain, the most commonly used strain in preclinical research today, is frequently used as a model organism for analysis of dopaminergic parameters implicated in human pathophysiology. Several components of the dopamine system have been shown to vary with age and sex, however knowledge of the contribution of these factors for dopamine release kinetics in the C57/Bl6J mouse strain is lacking. In the present study, we used an intracranial KCl-stimulation challenge paradigm to provoke release from dopaminergic terminals in the dorsal striatum of anaesthetized C57/Bl6J mice. By high-speed *in vivo* chronoamperometric recordings, we analyzed DA release parameters in male and female mice of two different ages. Our experiments demonstrate elevated DA amplitudes in adult compared to young mice of both sexes and higher DA amplitudes in females compared to males at both ages. Adult mice exhibited higher recovery capabilities after repeated stimulation than did young mice and also showed a lower variability in the kinetic parameters t_rise_ and t_80_ between stimulations. These results identified age- and sex- dimorphisms in DA release parameters and point to the importance of taking these dimorphisms into account when utilizing the C57/Bl6J mouse strain as model for neurological and neuropsychiatric disorders.

## Introduction

The dorsal striatum (DStr) is the largest input structure of the basal ganglia and it is heavily innervated by dopaminergic projections originating in the substantia nigra *pars compacta* (SNc) [1]. Coordinated release of dopamine (DA) into the DStr is crucial for the promoting the initiation of movement and degeneration of SNc DA neurons leads to bradykinesia, the main symptom of Parkinson's disease [2]. In addition to its role in motor control, dopaminergic signalling in the DStr is crucial for shaping habit formation [3], rendering its dysfunction important in a range of disorders including addiction [4].

The importance of the nigrostriatal DA system for human disorders has motivated substantial efforts in increasing the understanding of DA release kinetics by using experimental animals, primarily mice and rats. Spontaneous DA release occur tonically in a nanomolar range but in evoked responses, the release levels increase transiently to a micromolar range (reviewed in [5]). On a molecular level, the regulation of DA release and subsequent reuptake are interdependent processes that involve a range of different proteins with similar functions in rodents and humans. These include Tyrosine Hydroxylase (TH) which is the rate-limiting enzyme of catecholamine synthesis [6], the dopamine transporter (DAT), essential for DA reuptake as it translocates extracellular DA into the cytosol [7], and the Vesicular Monoamine Transporter 2 (VMAT2) which is responsible for packaging of DA into presynaptic vesicles [8]. Additional components including the DA metabolism machinery are also important for regulating DA release ability [9].

Studies of human DA release kinetics have shown that women have higher DA release levels than men [10]. Similar sex-dimorphism of DA release levels has been shown for rodents. Female Sprague-Dawley rats were shown by fast-scan cyclic voltammetry on brain slices to have higher DA release levels than males from the same strain [11]. Another study performed on superfused striatal tissue from CD-1 mice showed higher DA release levels upon potassium stimulation in females than males when analysed by HPLC [12]. Due to progressive alterations in the nigrostriatal DA system, age is also a confounding factor to consider when addressing DA release kinetics. For example, electrical stimulation of the medial forebrain bundle has revealed higher striatal DA release in adult than young male Wistar rats measured by fast cyclic voltammetry [13], findings supported by a study in male Sprague-Dawley rats which showed elevated levels in adult compared to young animals by microdialysis and HPLC [14]. The contribution of sex- and age-dimorphisms to DA release kinetics in the C57/Bl6J mouse strain often used as the reference mouse strain for physiological phenotypes [15] has not yet been addressed. In this study, we performed *in vivo* measurements of DA release and reuptake parameters in anaesthetized C57/Bl6J mice. In order to address the release and recovery capacity of the nigrostriatal DA system, we implemented a repeated KCl-stimulation paradigm in the DStr. KCl is known to evoke DA release amplitudes in the micromolar range [16], [17], comparable to the levels obtained upon electrical stimulation or administration of psychostimulants [11], [13], [18]. The recordings were performed using high-speed *in vivo* chronoamperometry, an established electrochemical method for second-by second level of temporal resolution and high chemical selectivity for DA [16], [19], [20]. *In vivo* recordings were performed in female and male C57/Bl6J mice of the young and adult age and all four groups of mice were compared to allow investigation of age- and sex-dependence in a range of DA release parameters.

## Materials and Methods

### Ethics statement

All mice used in the study were housed as previously described [17] in accordance with the Swedish regulation guidelines (Animal Welfare Act SFS 1998∶56) and European Union legislation (Convention ETS123 and Directive 2010/63/EU), and ethical approval was obtained from the Uppsala Animal Ethical Committee.

### Chronoamperometric in vivo measurement of DA release and reuptake

The experimental groups consisted of young (defined age 3–4 weeks, corresponding to postnatal days (P21-27) and adult (defined age 16–20 weeks) C57/Bl6J mice of both sexes according to: Young males (n = 7); Young females (n = 8); Adult males (n = 6) and Adult females (n = 8). Upon urethane anaesthesia (12.5%), each animal was placed in a digital stereotactic frame (World Precision Instruments, FL, USA) and kept at a body temperature of 37°C on a heating pad (Harvard apparatus, NH, USA). An incision was made over the skull; bregma and lambda were aligned using a dissection microscope (Nikon, Japan) together with the digital stereotactic frame. Anteroposterior ±0.07 mm difference between bregma and lambda was set as a threshold. Single carbon fiber electrodes (SF1A; 30 µm outer-diameter, 100–200 m length; Quanteon, LLC, KY, USA) were coated with 2–3 layers of a 5% Nafion solution (Sigma Aldrich, MO, USA) prior to use [21]. Working electrodes used for recordings displayed a limit of detection of less than 0.05 µM, a selectivity ratio exceeding 500∶1 over ascorbic acid and linear response to DA (*r* ^2^>0.995). A working electrode/micropipette unit was implanted according to a standard brain atlas [22]: anteroposterior, +1.1 mm, mediolateral, −1.5 mm and dorsoventral −3.2 mm from bregma. An Ag/AgCl reference-electrode was placed on the contralateral side.

**Figure 1:**
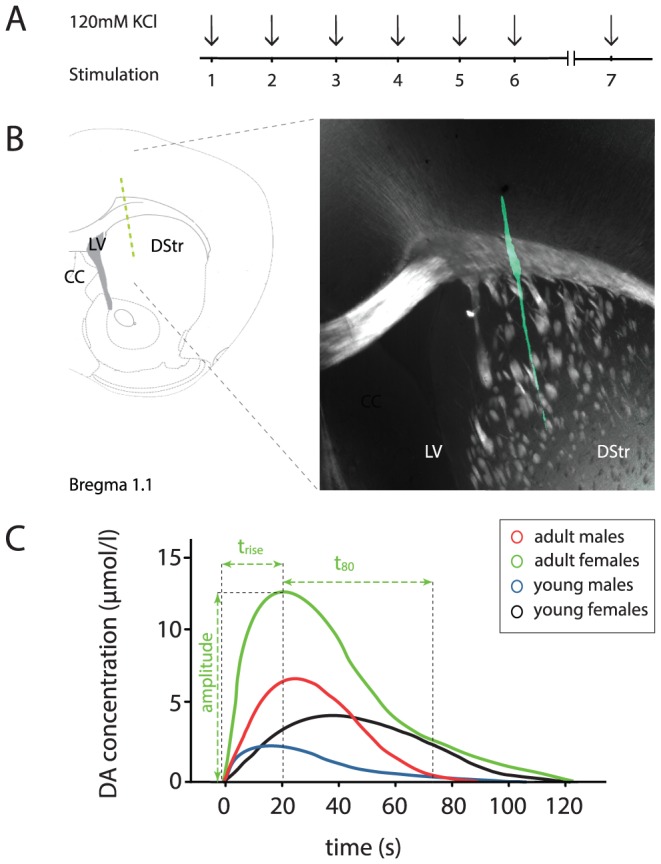
High-speed *in vivo* chronoamperometry recording setup and parameters. **A**: Experimental setup: 6 consecutive KCl-stimulations (1–6) in 2-minute intervals were followed by a 15-minute stimulation-free period upon which followed stimulation 7. Basal release capacity was defined by stimulation 1; recovery capacity was defined as a significant increase in DA amplitude upon stimulation 7 compared to stimulation 6; regain of basal release capability was defined as the absence of a significant difference in DA amplitude between stimulation 1 and stimulation 7. **B**: Left: Schematic illustration of a coronal brain section at Bregma 1.1 with recording electrode implicated by green dotted line. Right: Close-up of representative photomicrograph depicting the dorsal striatum (DStr) shown to the left as indicated by gray dotted lines; histological verification of the position of the DiO-coated carbon-fiber recording electrode (green) implanted in the DStr of a C57/BL6J mouse. **C**: Representative traces of DA release kinetics obtained by the high-speed chronoamperometry system following KCl-stimulation 1 for all four experimental groups: Green - adult females; Red - adult males; Black - young females; Blue - young males (shown in inset). Illustration of parameters analysed (green trace used as example): Amplitude, defined as the peak DA concentration (µM) from baseline; *t*_rise_, the time (seconds) between injection and maximum peak concentration; and t_80_, the time (seconds) from maximum peak concentration until 80% decrease of the maximum amplitude as a measure of DA clearance. CC; corpus callosum, LV; lateral ventricle.

High-speed *in vivo* chronoamperometry measurements were performed using the FAST16mkII recording system (Fast Analytical Sensing Technology, Quanteon, LLC, KY, USA). A square-wave pulse sufficient to oxidize DA was applied to the coated working electrode (applied, +0.55 V for 100 ms; resting, 0.0 V for 100 ms; with respect to the reference-electrode) and repeated at a frequency of 5 Hz. An average signal was automatically calculated for every 5 cycles by the recording software (Fast Analytical Sensing Technology, Quanteon, LLC, KY, USA) resulting in the temporal resolution of 1 data point per second (1 Hz). DA release was evoked by 7 pressure ejections (stimulations 1–7) of 100–120 nL of 120 mM KCl (Sigma Aldrich, MO, USA) applied locally (Picospritzer, Parker Hannifin, OH, USA). Stimulations 1–6 were applied in 2-minute intervals followed by a 15-minute resting period after which a final stimulation (7) was given (illustrated in Fig. 1A). This protocol was implemented to allow the analysis of basal release capacity (stimulation 1) as well as recovery and regain capacity. Recovery was hereby defined as a significant increase in DA amplitude upon stimulation 7 compared to stimulation 6. Regain of basal release capability was defined as the absence of a significant difference in DA amplitudes in stimulation 7 compared to stimulation 1. For verification of the implantation site, micropipettes were bathed in the fluorescent DiO cell labelling solution (Invitrogen, Thermo Fisher Scientific Inc, USA) prior to implantation. Upon sacrifice, brains were vibratome-sectioned (Leica VT1000S, Leica Biosystems, Germany) at 40 µm thicknesses (representative example, Fig. 1B). Images were captured on a Zeiss LSM 510 Meta confocal microscope and analyzed using Volocity software (Improvision, PerkinElmer, MA, USA).

### Data analysis and statistics

Redox ratios were calculated at the peak of every response to identify the analyte contributing to the electrochemical signal. DA typically displays a redox ratio of 0.7–0.9 *in vivo*, whereas possible interfering electrochemical species have lower redox ratios [21]. Only recordings with a redox ratio above 0.7 (average 0.76) were included in the data analysis. The following three parameters were examined: (1) amplitude, defined as the peak DA concentration (µM) from baseline; (2) *t*_rise_, the time (seconds) between injection and maximum peak concentration; and (3) t_80_, the time (seconds) from maximum peak concentration until 80% decrease of the maximum amplitude as a measure of DA clearance. All parameters are illustrated in Fig 1C which also shows one representative trace from each group.

To account for multiple comparisons, the data was analysed using ANOVA and post-hoc test with Bonferroni correction for stimulations 1–6. For comparison of DA amplitudes over time within each group, 1-way repeated measures ANOVA with KCl injection as repeated measure was applied. For analysis of stimulations 1-6 between each group, 3-way repeated measures ANOVA with sex and age as independent factors and KCl injection as repeated measure. Independently of this, we assessed recovery and regain upon 15-minutes of resting. For this, stimulations 1 and 6 as well as stimulation 6 and 7 were compared using a paired student's t-test.

To visualize each measurement value during initial release (stimulation 1) as well as before (stimulation 6) and after resting (stimulation 7) and to allow direct comparison between each group in a single figure, we computed scatter plot matrices. By doing so, we achieved simultaneous comparison of two variables (one on the x and one on the y axis) among all tested groups. Scatter plot matrices showing individual subject values were computed using a custom-made algorithm in Matlab (Mathworks, MA, USA).

Statistical analysis was performed in Graphpad Prism 6 (Graphpad Software, CA, USA) and R software (http://www.r-project.org/). A p-value equal to or less than 0.05 was considered significant (*p≤0.05, **p≤0.01, ***p≤0.001).

## Results

### DA release and reuptake parameters within adult and young groups of mice of both sexes

**Figure 2:**
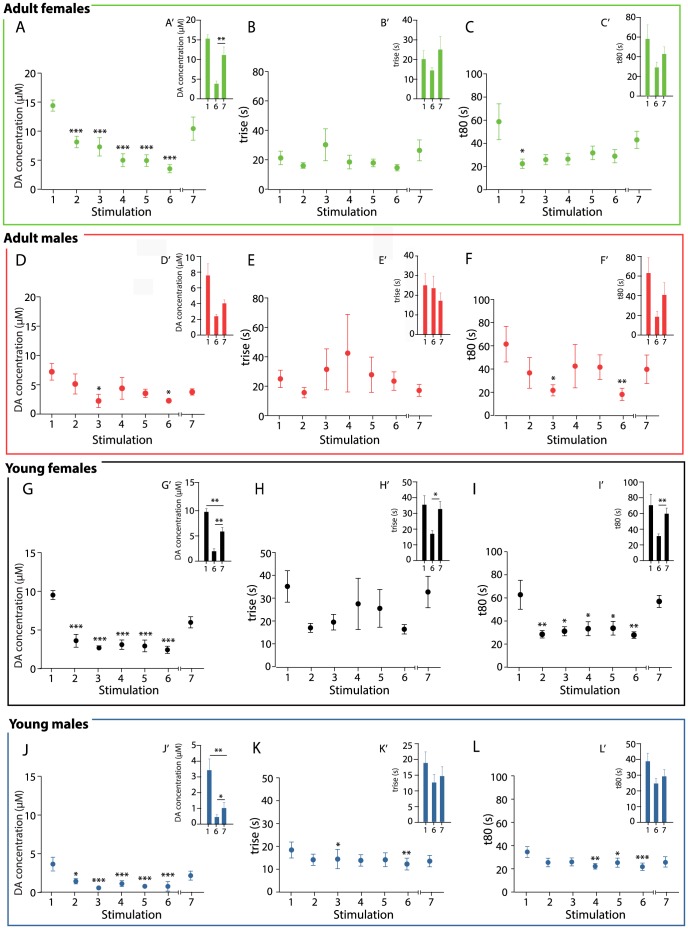
DA release and reuptake parameters within adult and young groups of mice of both sexes. Mean values obtained for each stimulation (1–7) within each group (Green - adult females (A–C); Red - adult males (D–F); Black - young females (G–I); Blue - young males (J–L) for parameters amplitude (A,D, G, J), *t*_rise_ (B, E, H, K) and t_80_ (C, F, I, L); Recovery capacity (stimulation 7 vs. 6) and regain of basal release capability (stimulation 7 vs. 1) shown as bar graph inserts for all groups and parameters (adult females (A′–C′); adult males (D′–F′); young females (G′–I′); young males (J′–L′). p<0.05: #, p<0.01: ##, p<0.001: ### as detected by 1-way repeated measure ANOVA, p<0.05: *, p<0.01: **, p<0.001: *** as detected by post-hoc test with Bonferroni correction (A-L) and paired student's t-test (A′-L′)

In adult females, a significant reduction of DA amplitudes was observed over the course of stimulations 1–6 with the initial KCl-stimulation (1) evoking a DA release amplitude of 15.33±1.02 µM and the last stimulation (6) an amplitude of 3.77±0.75 µM (1-way repeated measures ANOVA, stimulations 1 to 6, F(5,14) = 12.62, p<0.0001; post-hoc test with Bonferroni correction: stimulation 1 compared to stimulation 2: p<0.01; compared to stimulation 3,4,5,6: p<0.0001) (Figure 2 A). A resting period of 15 minutes led to a significant increase in DA amplitude to 11.10±2.12 µM (paired student's t-test, p<0.01, stimulation 7 compared to 6) and was sufficient for adult females to recover to initial release levels (paired student's t-test, p = 0.0783, stimulation 7 compared to 1) (Figure 2 A′).

No significant alterations were seen in t_rise_ during stimulation 1 to 6 (1-way repeated measures ANOVA, stimulations 1 to 6, F(5,24) = 1.16, p = 0.3789) (Figure 2 B) or upon stimulation (7) after 15-minutes of resting (paired student's t-test stimulation 1 compared to 7: p = 0.0922; stimulation 7 compared to 6: p = 0.4866) (Figure 2 B′). The parameter t_80_ was significantly decreased only following stimulation 2 compared to stimulation 1, but not during stimulations 3,4,5 or 6 (1-way repeated measures ANOVA, stimulations 1-6, F(5,31) = 2.89, p<0.05; post-hoc test with Bonferroni correction: stimulation 1 compared to stimulation 2: p<0.05) (Figure 2 C). Stimulation 7 after 15-minutes of resting did not led to altered t_80_ compared to initial values (paired student's t-test, p = 0.4866, stimulation 7 compared to 1) or stimulation 6 (paired student's t-test, p = 0.0922) (Figure 2 C′). Taken together, within the adult female group, repeated stimulation led to a gradual decrease in DA amplitude so that upon stimulation 6, DA amplitudes were only 25% as high as upon stimulation 1. 15 minutes of resting were sufficient for adult females to regain basal release capabilities. Even though DA amplitudes decreased due to repeated stimulation, this did not affect rise time. Reuptake time was significantly lower in the second stimulation compared to the first, but not altered upon the following stimulations.

In adult males, the repeated stimulation in 2-minute intervals led to a significant reduction in DA amplitudes (1-way repeated measures ANOVA, stimulations 1 to 6, F(5,14) = 3.85, p<0.05; post-hoc test with Bonferroni correction: stimulation 1 compared to stimulation 3: p<0.01) (Figure 2 D). DA amplitude in response to the initial stimulation was 7.76±1.52 µM that decreased to a minimum of 2.43±1.22 µM after stimulation 3. No recovery occurred after 15 minutes of resting, (stimulation 7: 2.46±0.25 µM compared to stimulation 6: 4.07±0.58 µM) (paired student's t-test, p = 0.0585). Release levels after resting (stimulation 7) were not significantly lower than following the initial stimulation (paired student's t-test, p = 0.0687, stimulation 7 compared to 1) (Figure 2 D′).

The parameter t_rise_ did not significantly differ between stimulation 1 to 6 (1-way repeated measures ANOVA, stimulations 1 to 6, F(5,31) = 0.24, p = 0.9418) (Figure 2 E). Resting did not alter t_rise_ in response to stimulation 7 in comparison to stimulation 1 (paired student's t-test, p = 0.2070) and 6 (paired student's t-test, p = 0.3672) (Figure 2 E′). In contrast, t_80_ significantly decreased over the course of stimulation 1 to 6 (1-way repeated measures ANOVA, stimulations 1 to 6, F(5,18) = 4.64, p<0.01; post-hoc test with Bonferroni correction: stimulation 1 compared to 3: p<0.05; compared to stimulation 6: p<0.01) (Figure 2 F). The parameter t_80_ was not significantly reduced after 15-minutes of resting in response to stimulation 7 compared to initial values (paired-student's t-test, p = 0.1570, stimulation 7 compared to 1) and had not increased compared to stimulation 6 (paired-student's t-test, p = 0.1712) (Figure 2 F′). Thus, within the adult male group, DA amplitudes decreased due to repeated stimulation to a minimum of 31% of initial values (upon stimulation 3 and 6) and recovered to basal levels after 15 minutes of resting. Rise time was independent of the amount of DA released during the stimulation while reuptake time was significantly lower in the stimulations where least DA was released (stimulation 3 and 6).

In young females, KCl stimulation evoked an initial DA release of 9.55±0.63 µM which decreased significantly over the course of stimulation 1 to 6 reaching a minimum of 1.88±0.50 µM after stimulation 6, directly prior to resting (1-way repeated measures ANOVA, stimulations 1 to 6, F(5,22) = 9.028, p<0.0001; post-hoc test with Bonferroni correction: stimulation 1 compared to stimulation 2: p<0.01; compared to stimulation 3,4,5,6: p<0.001) (Figure 2 G). Following 15 minutes of resting, stimulation 7 resulted in an increase of DA amplitude to 5.72±0.78 µM (student's t-test p<0.01 stimulation 7 compared to 6). However, release levels following resting (stimulation 7) were still significantly lower than in response to the first KCl ejection (paired student's t-test, p<0.01, stimulation 7 compared to 1) (Figure 2 G′).

No differences in t_rise_ were seen during stimulation 1 to 6 (1-way repeated measures ANOVA, stimulations 1 to 6, F(5,35) = 2.24, p = 0.0721) (Figure 2 H). Following resting, however, t_rise_ for stimulation 7 was significantly elevated in comparison to stimulation 6 (paired student's t-test, p<0.05). No difference was detected between stimulation 1 and 7 (paired student's t-test, p = 0.8523) (Figure 2 H′). The parameter t_80_ was significantly decreased in stimulations 2 to 6 compared to the first (1-way repeated measures ANOVA, stimulations 1 to 6, F(5,22) = 6.83, p<0.001; post-hoc test with Bonferroni correction: stimulation 1 compared to stimulation 3,4,5: p<0.01; compared to stimulation 2,6: p<0.001) (Figure 2 I). Following stimulation 7, t_80_ was significantly increased compared to prior resting (paired student's t-test, p<0.01. stimulation 7 compared to 6), and in the range of the initial stimulation (paired student's t-test, p = 0.5827, stimulation 7 compared to 1) (Figure 2 I′). Hence, within the young female group, DA amplitudes decreased gradually, reaching a minimum of 20% of basal release upon stimulation 6. While 15 minutes of resting led to a significant recovery of DA amplitudes, young females did not regain basal release capabilities during this time. Reuptake time was significantly higher upon the initial release and stimulation 7 than upon the other stimulations. Repeated stimulation and reduced DA amplitudes did not significantly alter rise time.

In young males, the DA amplitudes decreased significantly during the repeated stimulation in 2-minute intervals (1-way repeated measures ANOVA, stimulation 1 to 6, F(5,8) = 16.59, p<0.0001; post-hoc test with Bonferroni correction: stimulation 1 compared to stimulation 2,3,4,5,6: p<0.0001) (Figure 2 J). Initial release levels in this group were at 3.40±0.72 µM, decreasing to a minimum of 0.47±0.15 µM after stimulation 6. During stimulation 7, the amplitude was with 1.04±0.36 µM significantly higher (paired student's t-test p<0.05, stimulation 7 compared to 6) but significantly lower than following the initial stimulation (paired student's t-test, p<0.01, stimulation 7 compared to 1) (Figure 2 J′).

The parameter t_rise_ decreased significantly over the course of stimulations 1 to 6 (1-way repeated measures ANOVA, stimulations 1 to 6, F(5,34) = 4.04, p<0.01; post-hoc test with Bonferroni correction: stimulation 1 compared to 3: p<0.05; compared to stimulation 6: p<0.01) (Figure 2 K). After resting, t_rise_ was not significantly altered in comparison to stimulation 1 (paired student's t-test, p = 0.1617) or stimulation 6 (paired student's test, p = 0.2425) (Figure 2 K′). Also, the parameter t_80_ decreased significantly over the course of the consecutive stimulations (1-way repeated measures ANOVA, stimulations 1 to 6, F(5,14) = 5.41, p<0.01; post-hoc test with Bonferroni correction: stimulation 1 compared to 5: p<0.05; compared to stimulation 4: p<0.01; compared to stimulation 6 p<0.001) (Figure 2 L). Upon stimulation 7, t_80_ was not significantly altered compared to stimulation 1 (paired student's t-test p = 0.0940) or stimulation 6 (paired student's t-test p = 0.0696) (Figure 2 L′). In summary, within the young male group, DA amplitudes decreased due to repeated stimulation reaching a minimum of 14% of basal release in stimulation 6. 15 minutes of resting led to a recovery of DA amplitudes but were not sufficient for young males to regain initial DA release capabilities. Reuptake time decreased upon repeated stimulation and rise time was significantly lower than upon stimulation 1 when DA amplitudes were smallest (stimulation 3 and 6).

### Comparing DA release and reuptake parameters between adult and young groups of mice of both sexes

**Figure 3:**
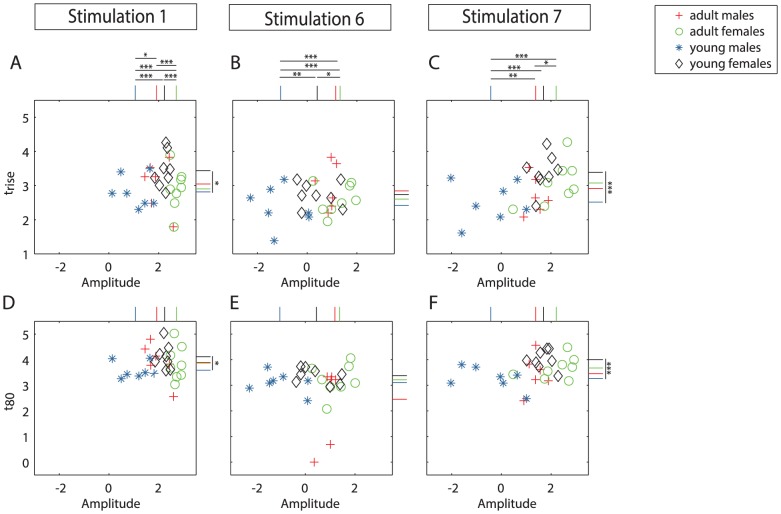
Comparison of DA release and reuptake parameters between adult and young groups of mice of both sexes. Scatter plots of DA amplitude vs. t_rise_ (A–C) and DA amplitude vs. t_80_ (D–F) as logarithmic values (natural logarithm) for each individual mouse after stimulation 1 (A, D), 6 (B, E) and 7 (C, F), respectively. Same color-coding as in Fig. 1 and 2: Green - adult females; Red - adult males; Black - young females; Blue - young males. Bars on the side of each plot depict mean values of the groups upon each specific stimulation (1, 6 and 7) and stars indicate statistically significant differences between indicated groups. p<0.05: *, p<0.01: **, p<0.001: *** as detected by 3-way repeated measures ANOVA and post-hoc test with Bonferroni correction.

Following the analysis within the groups, we compared DA amplitudes as well as the kinetic parameters t_rise_ and t_80_ between all four groups of mice. Among adult mice, females showed higher DA amplitudes than males (3-way repeated measure ANOVA, stimulations 1 to 6, F(31,6) = 9.88, p<0.01). Following stimulation 1, the amplitude in adult females was twice as high as in adult males (Post-hoc test with Bonferroni correction: p<0.001) (Figure 3 A) while no significant difference was detected upon stimulation 6 (Post-hoc test with Bonferroni correction: p = 0.950) (Figure 3 B). Upon stimulation 7 after 15-minutes of resting, the mean DA amplitude in adult females was 173% higher than in adult male mice (paired student's t-test, p<0.05) (Figure 3 C). Neither t_rise_ (Figure 3 A–C) nor t_80_ (Figure 3 D–F) exhibited differences between the two experimental groups.

Similar as observed for adult mice, also among young mice, females exhibited higher DA release than males (3-way repeated measure ANOVA, stimulations 1 to 6, F(31,7) = 7.23, p<0.01; Post-hoc test with Bonferroni correction: stimulation 3,5: p<0.05; stimulation 6: p<0.01; stimulation 1: p<0.001). Following stimulation 1, the DA amplitude of young males was only 36% of that of young females (Post-hoc test with Bonferroni correction: p<0.001) (Figure 3A). In contrast to the adult groups, however, in young animals, the difference in DA amplitudes even increased after stimulation 6. Young females reached DA amplitude that was 303% higher than in young males (Post-hoc test with Bonferroni correction: p<0.01) (Figure 3 B). As young female but not young male mice exhibited recovery during 15 minutes of resting, females released 452% more DA during stimulation 7 compared to young males (paired-student's t-test, p<0.001) (Figure 3 C).

Following the initial stimulation (1), young females possessed a significantly higher t_rise_ (3-way repeated measure ANOVA, stimulations 1 to 6, F(31,8) = 4.44, p<0.05, Post-hoc test with Bonferroni correction: stimulation 1: p<0.05) (Figure 3 A) and t_80_ (3-way repeated measure ANOVA, stimulations 1 to 6, F(31,6) = 8.96, p<0.01; Post-hoc test with Bonferroni correction: stimulation 1: p<0.05) (Figure 3 D) than did young males, a difference that remained after stimulation 7 (paired-student's t-test; t_rise_: p<0.05, t_80_: p<0.01) (Figure 3 C and F). In summary, DA amplitudes were generally larger in female animals compared to males of the same age.

When comparing mice of the same sex, higher DA amplitudes were detected in adult than young mice (males: 3-way repeated measure ANOVA, F(31,5) = 12.03, p<0.01; females: 3-way repeated measure ANOVA, stimulations 1 to 6, F(31,7) = 22,59, p<0.0001). Following stimulation 1, young males reached 44% of the amplitude displayed by the adult males (Post-hoc test with Bonferroni correction: p<0.05) while young females reached 62% of the maximal amplitude of adult females (Post-hoc test with Bonferroni correction: p<0.01). Similar differences were detected upon stimulation 6 and 7 in both males (Post-hoc test with Bonferroni correction: stimulation 6: p<0.05; stimulation 7: p<0.001) and females (Post-hoc test with Bonferroni correction: stimulation 6: p<0.05; stimulation 7: p<0.05) (Figure 3 B and C). Interestingly, this age-dependent difference within each sex was only observed in the amount of DA released. Both t_rise_ (Figure 3 A–C) and t_80_ (Figure 3 D–F) were largely similar in adult and young animals of each sex. Further, the DA amplitude of adult males was in a similar range as young females for all experimental time points (3-way repeated measure ANOVA, F(31,1) = 5.58, p = 0.3255) while adult females exhibited significantly higher DA amplitudes than young male animals (3-way repeated measure ANOVA, stimulations 1 to 6, F(31,8) = 27.97, p<0.0001 (Figure 3 A-C).

In summary, DA amplitudes were generally larger in adult than young mice of the same sex. The lower DA amplitudes in males compared to females led to the fact that adult males and young females exhibited fairly similar amplitudes while the difference was highest between adult females and young males. Adult animals of both sexes, but not young animals of any sex, were able to regain basal release capabilities upon 15 minutes of resting. DA amplitudes were significantly increased in stimulation 7 compared to stimulation 6 in all groups, showing that all groups recover upon resting.

## Discussion

In this study, we used an intracranial challenging paradigm in the DStr of anaesthetized C57/Bl6J mice. An initial dose of KCl (stimulation 1) was followed by additional KCl-ejections in 2-minute intervals (stimulation 2–6). The experiment was finalized by a seventh stimulation after a 15-min long resting period to allow analysis of capacity for recovery and capacity to regain initial release levels. KCl was previously shown to elicit DA release in a µM-range, comparable to electrical stimulation or psychostimulants [11], [13], [18]. High release levels were of particular interest here as we wished to assess the full capacity of the striatal DA system. Analysis by high-speed chronoamperometry revealed elevated DA amplitudes in adult compared to young mice of both sexes. Furthermore, young and adult females showed higher DA release levels as well as heightened capability for recovery compared to male mice of corresponding ages. Such age- and sex-dimorphisms have not been described before for the C57/Bl6J mouse strain, generally considered the reference strain for physiological phenotypes [15]. A study performed in mice to directly address sex differences in basal DA release capacity of mice corroborates our findings. By HPLC analysis, superfused striatal tissue fragments from CD-1 mice showed higher DA release levels in females than males after potassium stimulation [9]. Results from DA release studies in rats of various strains are also in line with our findings. Adult male rats have been shown to release larger amounts of DA upon electrical stimulation and to have a faster reuptake speed than young males [13], while adult female rats show elevated release levels compared to males of same age [11].

DA reuptake is mainly mediated via the electrogenic nature of DAT [23], and in addition to age-dependent differences in DA amplitudes, our data suggest age-dependence in DA clearance. Adult mice managed to achieve higher DA amplitudes than did young animals without increasing t_80_, indicating a more efficient reuptake machinery with increased age. Importantly, sustained depolarization, as in the case of the applied potassium used here, could interfere with DAT function and hence affect the clearance. However, as the amount of KCl used for stimulation is equally high in all animals of this study, possible effects on DAT function should be similar in all animals, thus preserving the intrinsic differences between the groups.

Further, rise time (t_rise_) was not significantly different between C57/Bl6J males and females even though the total amount of DA released varied greatly throughout the groups and individual stimulations. Based on the constant rise time with varying DA amplitudes in both adult males and females, it seems that DA release velocity is adjusted to allow for exact timing of release independently of the actual amount of DA released. In young males, t_rise_ was shorter in stimulations 3 and 6, where DA amplitudes lowest. Throughout stimulation 1 to 6, t_rise_ in young males significantly decreased with decreasing DA amplitude. In a similar manner, t_rise_ increased significantly in young females following resting and was higher in young females compared to young males during initial stimulation and after resting, when their DA release levels are at their highest. Thus, young mice seem to be unable to uphold the precise timing of DA release seen in adult mice during varying DA amplitudes.

In any repeated challenging paradigm, newly produced or recycled DA should be loaded into synaptic vesicles for release following the upcoming stimulation. Interestingly, 20-minute, but not 2-minute, intervals between stimulations were previously shown to be sufficient for recovery of DA amplitudes in adult male rats [24]. In our study, repeated stimulation in 2-minute intervals indeed led to a decline in DA release in both male and female animals. A 15-minute resting period resulted in a significant recovery of DA amplitudes in both adult and young female mice, while male mice of both age groups failed to do so. VMAT2, the transporter responsible for vesicular packaging of DA in nerve cells, has been shown to be more efficient in female than male CD1 mice [18]. Based on this previous finding, the capability of female mice to replenish the pool of DA-loaded vesicles can be expected to be higher, potentially leading to the herein observed higher DA concentrations after resting. In addition to sex differences in VMAT2 function resulting in more efficient packaging in females [18], sex-dimorphism has also been described for several other components of the dopaminergic system [25]. Human single photon emission computed tomography (SPECT) studies have shown that females have a higher number and density of DAT [26] as well as higher DA synthesis rates [27] and elevated DA release [10]. This phenomenon has been used to explain why women seem less vulnerable to certain neurological conditions such as schizophrenia [28], OCD [29], Tourette's syndrome [30] and Parkinson's disease [31], all of which are disorders that involve the striatal dopaminergic system.

Taken together, the age- and sex-dependent differences in DA release and reuptake capacity observed in our study have several implications for future studies utilizing C57/Bl6J mice to explore the nigrostriatal DA system. Any study carried out without consideration for age or sex could suffer from the group differences identified here. For example, comparing two experimental groups where one randomly consists of mostly relatively young male mice and the other mainly of older female mice, i.e. the two groups exhibiting the highest difference from each other in terms of basal DA amplitudes, could have severe consequences on the interpretation of the data obtained. Further, considering the finding that rise time and clearance differ in young but not adult mice, age differences within an experimental setup of mice have to be considered when performing studies that include manipulations of the DA kinetics. Finally, in view of the finding that DA amplitudes decrease rapidly in response to a potent challenge and young mice seem to need longer resting time for their DA system to regain its initial capacities, any treatment that causes a strong voiding of DA contents should take age into account. In summary, C57/Bl6J is the most commonly used inbred laboratory mouse strain. Data presented here might serve to increase our awareness of sex- and age influences on the release and reuptake capacity of the DA system within this strain which is frequently used as genetic background for the wide array of transgenic mouse lines utilized to model neurological and neuropsychiatric disorders.
